# Giant Cell Tumor with Secondary Aneurysmal Bone Cyst of the Patella: A Case Report

**DOI:** 10.7759/cureus.5819

**Published:** 2019-10-01

**Authors:** Sujit K Tripathy, Sunil Doki, Gayatri Behera, Mukund Sable

**Affiliations:** 1 Orthopaedics, All India Institute of Medical Sciences, Bhubaneswar, IND; 2 Pathology, All India Institute of Medical Sciences, Bhubaneswar, IND

**Keywords:** aneurysmal bone cyst, benign bone tumor, patella, giant cell tumor, osteoclastoma, bone cyst

## Abstract

A 15-year-old girl presented with pain and swelling on the anterior aspect of the right knee for one year. The radiological evaluation with x-rays and magnetic resonance imaging suggested a benign aggressive lesion of the right patella with a cortical breach. Core needle biopsy of the lesion revealed it to be a giant cell tumor (GCT). She was treated with total patellectomy and end-to-end repair of quadriceps to the patellar tendon. The histopathological report of the whole specimen revealed it to be a GCT with secondary aneurysmal bone cyst (ABC). After 24 months, she was asymptomatic, and there was no evidence of local recurrence or distal metastasis. An extensive review of the literature revealed only four cases of combined GCT with secondary ABC in the patella. Though rare, GCT with secondary ABC of the patella should be kept as a differential diagnosis for anterior knee pain and swelling in young patients. The diagnosis is solely based on histopathological findings. It is imperative to obtain a precise tissue diagnosis in the preoperative period to plan appropriate treatment.

## Introduction

Giant cell tumor (GCT) accounts for 4%-5% of all primary bone tumors, and it is usually seen at the end of long bones around the knee after skeletal maturity. However, there are few case reports of unusual location and in a few cases even in sesamoid bones such as patella [1]. Primary patellar tumors are rare; in a series of 7,975 primary bone tumors, Dahlin and his co-workers reported only 10 (0.12%) patellar lesions [2]. A majority (70-90%) of the patellar tumors are benign. The most common tumor is GCT (33%) followed by chondroblastoma (16%) [1,3-5]. A combined GCT and aneurysmal bone cyst (ABC) is extremely rare in the patella, and only four case reports are available in medical literature till date [6-9]. We hereby submit a case of patellar GCT with secondary ABC in a young female who presented with pain and swelling on the anterior part of the knee. We have taken due consent from the patient regarding the publication of this case.

## Case presentation

**Figure 1:**
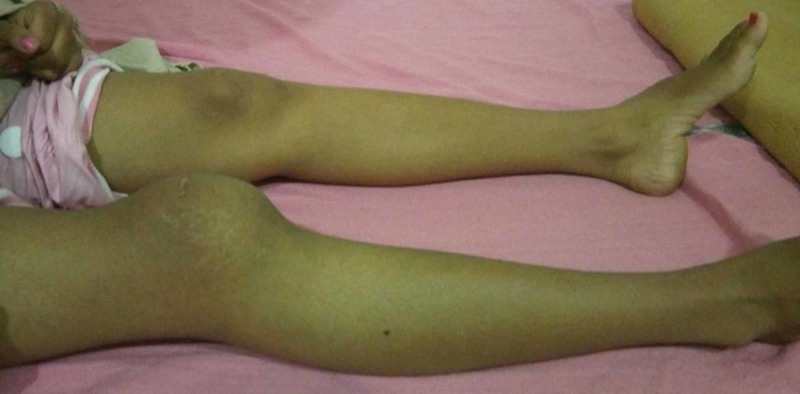
Clinical picture of the patient showing swelling of the right knee

A 15-year-old girl was presented with complains of pain and swelling in the right knee for the last one year. She was limping for the last six months with gradual progression of symptoms. There was no trauma history, fever, weight loss, loss of appetite or history of exposure to tuberculosis. Examination revealed that the swelling was anterior and was arising from the underlying patella bone (Figure 1). The swelling was tender to deep pressure. There was no localized warmth, synovial thickening, effusion or skin adhesion over the swelling. The range of motion of her knee was 0 to 60 degrees with terminal flexion being painful. There was no ligamentous instability in the knee, and there was no distal neurovascular deficit. 

**Figure 2:**
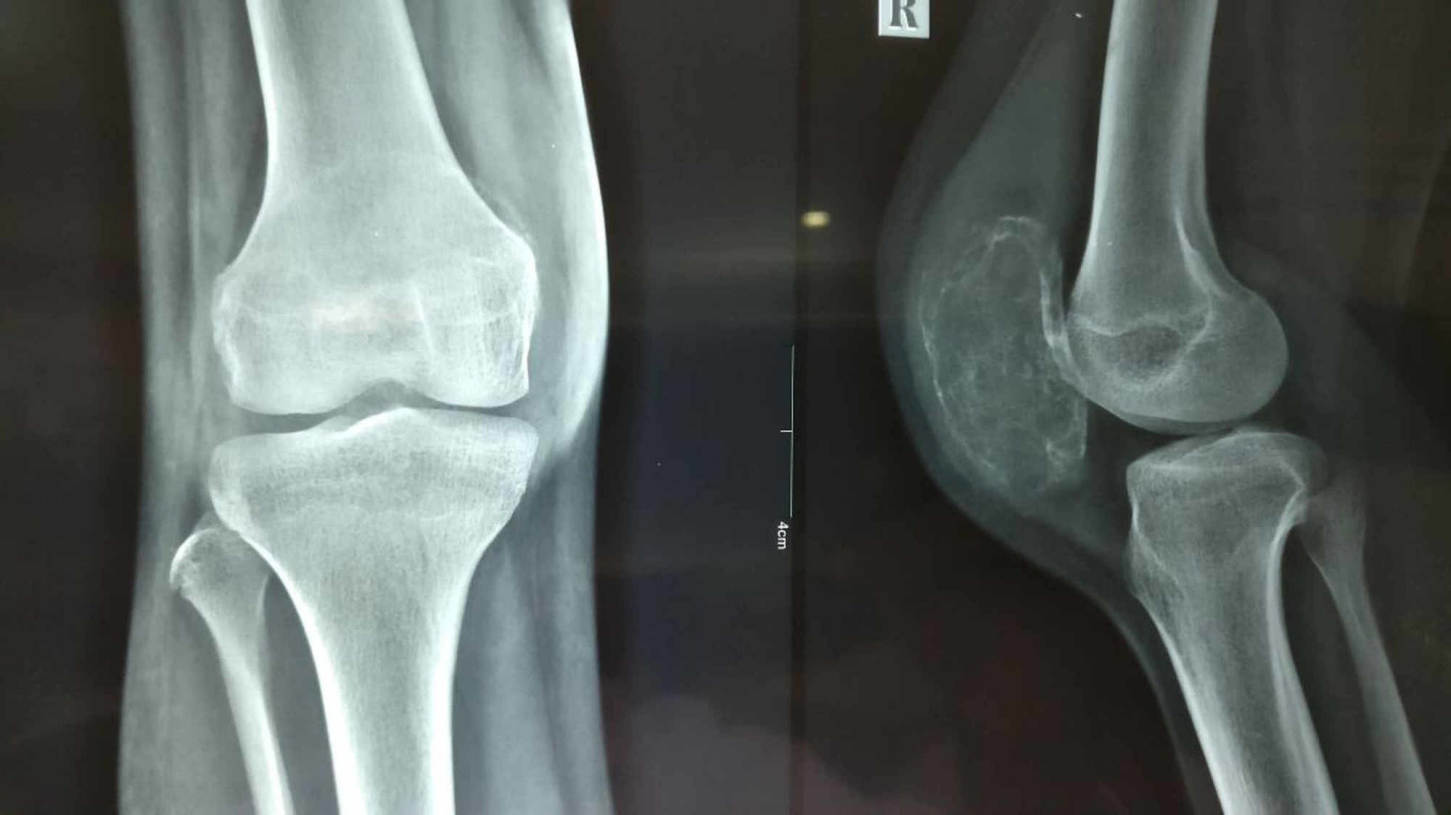
Radiograph of the right knee showing expansile lytic lesion of the whole patella

**Figure 3:**
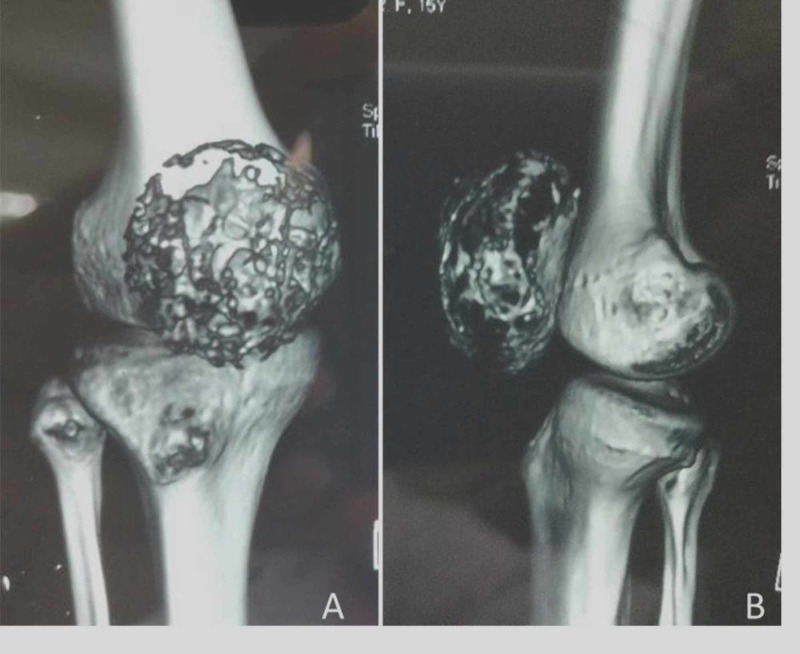
Computed tomography scan shows complete involvement of whole patella with no soft tissue extension

**Figure 4:**
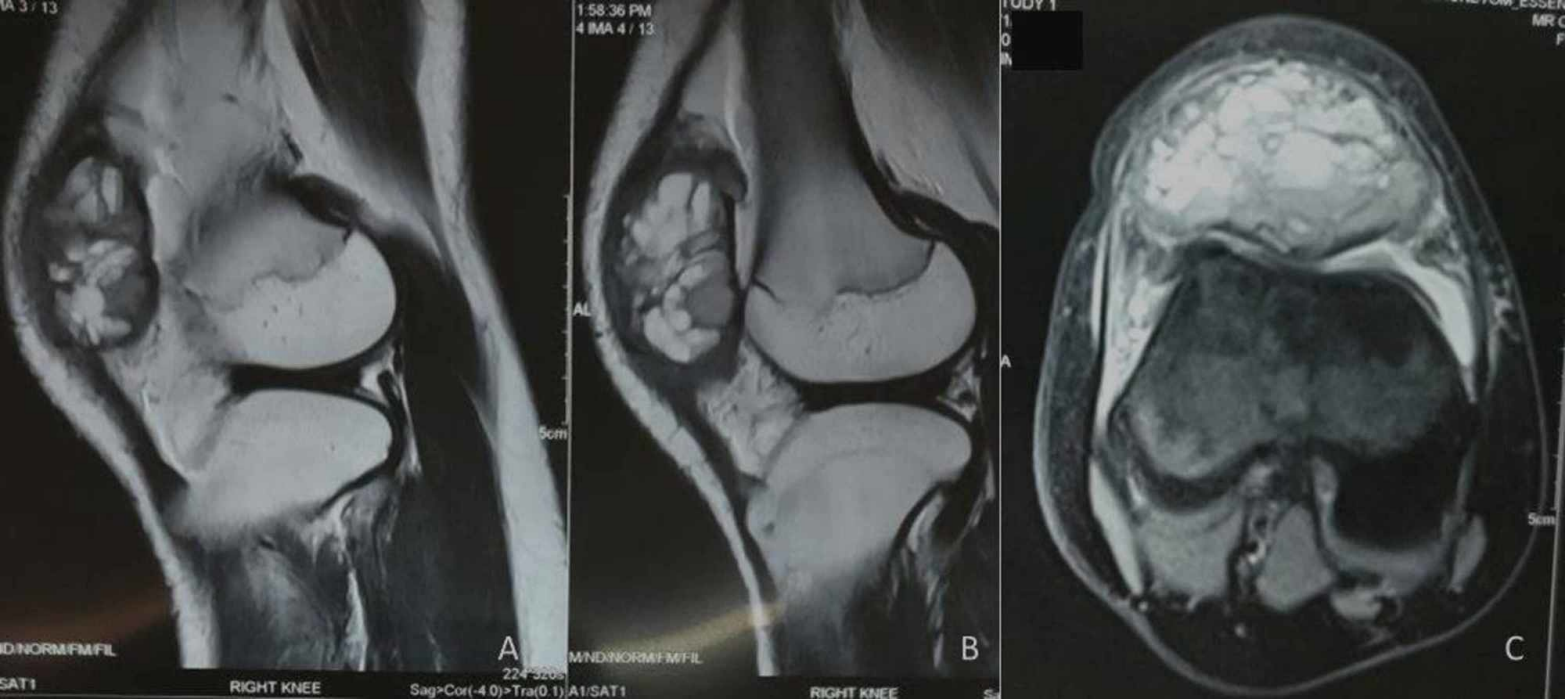
Sagittal (A, B) and axial cut sections show T2-weighted hyperintense signal with no involvement of overlying soft tissue

 Laboratory studies revealed hemoglobin of 11.4gm/dl and white cell count of 9,700 cells/mm^3^. Differential counts were within normal range - ESR was 20, C-reactive protein was negative, and alkaline phosphate was within normal limits. Mantoux test was non-reactive. Radiographs of the right knee showed lytic and expansile lesion involving the entire patella with thinned out cortex in a diffuse pattern suggesting a benign lesion (Figure 2). There was no periosteal reaction. Computed tomography scan clearly delineated the lesion (Figure 3). The lesion on magnetic resonance imaging (MRI) was T2-hyperintense with small multiple fluid pockets. There were areas with hemorrhagic component and cortical breach (Figure 4). Based on these clinic-radiological findings, a diagnosis of GCT of the patella was made with a differential diagnosis of ABC and telangiectatic osteosarcoma. A core needle biopsy revealed it to be GCT of the patella. The patient was searched for other metastatic foci in the chest, but her chest x-ray and CT scan were normal.

**Figure 5:**
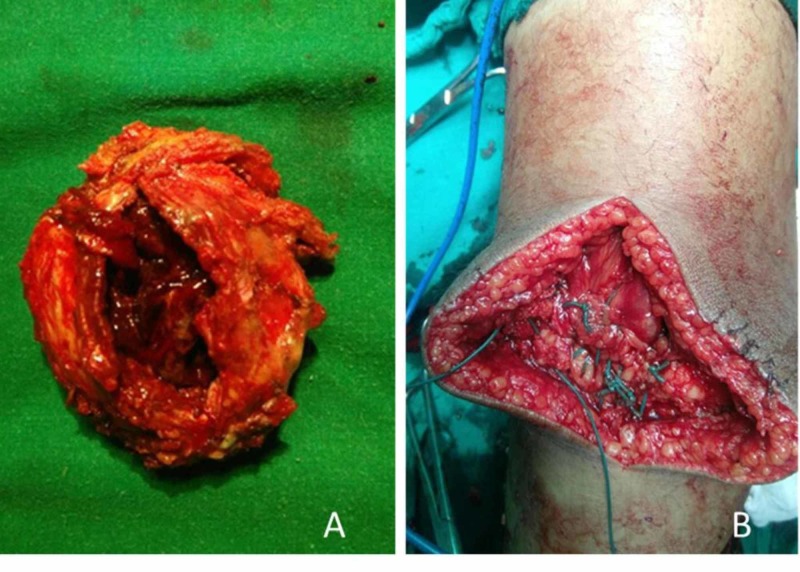
A-Excised specimen of patella shows blood filled cavity with in the patella and occasional break in cortex, B- Extension mechanism repair by tying Quadriceps tendon to patellar tendon with Ethibond No 5 suture

**Figure 6:**
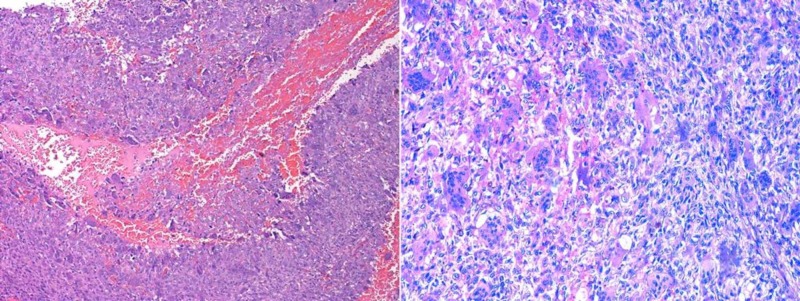
Microscopic picture (normal and magnified view) of excised specimen of patella shows spindle-shaped mononuclear cells with osteoclastic giant cells. Foci of hemorrhage, hemosiderin deposits and aggregates of histiocytes were found confirming the diagnosis of aneurysmal bone cyst secondary to giant cell tumor

 Total patellectomy with extensor repair was planned. Intraoperatively, it was found that the whole patella was involved, and the cortical structure was so thinned out that it could be deformed with slight pressure from a finger leading to overall destruction of the patella. There was no invasion of surrounding soft tissue (Figure 5A). The patient underwent total patellectomy and extensor mechanism was repaired with an end-to-end repair of quadriceps tendon to patellar tendon (Figure 5B). Intraoperatively, the flexion of the knee was full. The whole specimen was sent for histopathological study. Microscopically the tumor consisted of spindle-shaped mononuclear cells with osteoclastic giant cells. The foci of hemorrhage, hemosiderin deposits, and aggregates of histiocytes were found confirming the diagnosis of aneurysmal bone cyst secondary to giant cell tumor (Figure 6).

**Figure 7:**
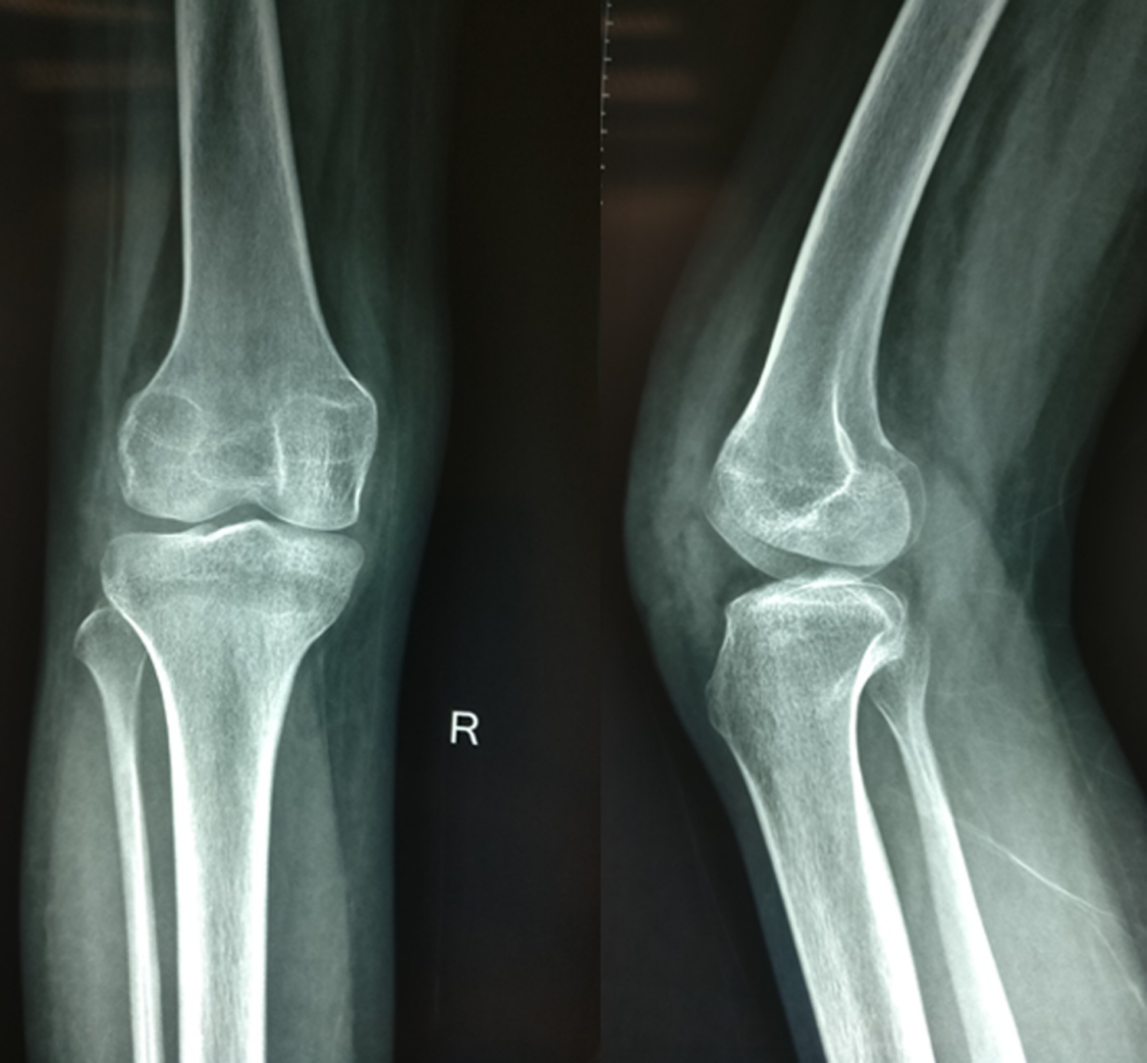
Follow up radiograph at 2-years shows absence of patella with no signs of recurrence

 In the postoperative period, the limb was splinted with a posterior slab for 4 weeks to prevent knee flexion, and then gradual knee flexion was started. At the end of six months, the patient had a complete range of motion (0 to 130 degree) with a normal gait, and there were no complaints. On subsequent follow up at 24-months, there were no clinical or radiological signs of local recurrence (Figure 7) or distant metastasis.

## Discussion

The overall incidence of ABC is 1.4% and typically seen in long bones and spine of children and young adults [10-13]. About 70% of ABCs are primary and arise de novo. Secondary ABCs (30%) are normally associated with GCT, chondroblastoma, osteoblastoma, non-ossifying fibroma, fibrous dysplasia, chondromyxoid fibroma, eosinophilic granuloma, or osteosarcoma [10-13]. The association of secondary ABC has been seen in 14% of GCT [1,10-13]. Although GCT and ABC are common benign tumors of the patella, a combined GCT and ABC case is extremely rare [6-9].

The clinical presentation of GCT with secondary ABC is the same as that of isolated GCT in patella [6-9]. Pain, swelling, and restriction of movement may be there in any lesion of the patella; hence, radiological and histological evaluations are crucial. It has been reported that only 20% of secondary ABCs have a typical radiologic appearance and in 80% of the cases, the main tumor dominates the radiological picture [14]. Unless the fliud level is visualized on MRI, it is difficult to suspect ABC. Overall the diagnosis relies on histopathology only. Again, on biopsy, if the material is aspirated from the cystic space, it may reveal hemorrhage and mislead the diagnosis or point towards ABC only [7,9]. Hence, multiple specimens from cystic as well as solid areas of tumors are essential to confirm the diagnosis from histopathology. Low et al. and Yo et al. have warned that a small biopsy and limited sample may be mischievous in combined ABC and GCT [7,9].

 Although the combined GCT and ABC may sound interesting, ABC has no implications as far as the management or the outcome of treatment of GCT is concerned [6-9]. However, it is surprising to see some of the previous reports in which the authors have directly proceeded for surgery without doing a tissue diagnosis in the preoperative period [7-8]. Song et al. and Yu et al. cited many differential diagnoses (GCT, ABC, telangiectactic osteosarcoma, and giant cell reparative granuloma) based on clinical and radiological findings, but they all directly went for surgery without preoperative or intraoperative tissue diagnosis [7-8]. The role of tissue diagnosis using a core needle biopsy can neither be overlooked nor underestimated [15]. This process is minimally invasive and provides a clue to the operating surgeon preoperatively. The most important benefit is that it can exclude malignant pathology in case the treatment is different. Low et al. and Marudanayagam et al. performed the intraoperative frozen section and preoperative core needle biopsy respectively to establish the diagnosis [6,9]. However, both these authors stressed on the avoidance of limited sample or small biopsy as in both of their cases they could diagnose only ABC and missed the GCT. Great caution must be taken while performing core needle biopsy preoperatively as there is a high chance of taking the material only from the cystic space which may erroneously give the result of ABC. The fear is that missing a concomitant malignant pathology in ABC may have grave consequence. Solid and cystic material must be obtained at various sites of the lesion to a have proper preoperative diagnosis. There must be a preoperative precise tissue diagnosis in patellar lesion to plan appropriate treatment. 

 In the previously reported cases, the area of involvement was not much extensive. The authors had curated the lesion and filled up the defect with a bone graft or bone graft substitute or cement [6-9]. They all have reported excellent outcome with no evidence of recurrence. In our case, the lesion was aggressive with a cortical breach, and there was overall destruction of the patella, so we preferred total patellectomy and reconstruction of the extensor mechanism. Complete patellectomy has been recommended in some of the reports of isolated GCT patella. The outcome of complete patellectomy needs to be assessed from the reports as it is the extreme form of the disease for which such treatment was planned. Again, it involves complete removal of the patella and the extensor mechanism repair; hence, the functional outcome may be different or compromised as compared to another relatively simple approach such as curettage and filling up the defect. Only one case of total patellectomy has been reported in GCT of the patella. Malhotra et al. reported a case of aggressive GCT treated with total patellectomy and extensor mechanism repair using a patellar allograft [16].

 GCT lesions are locally aggressive with high chances of recurrence rate. It has been reported that the incidence of metastasis is up to 1%-2% [17-19]. To exclude metastasis, we evaluated the case with chest x-ray and CT scan. Many authors report that GCT recurrence is high during the initial two years [17-19]. At 24-months of surgery, the patient was free of symptoms; there was no radiological evidence of recurrence or distant metastasis. Even then a long-term follow-up is essential and the patient is still under our follow-up.

## Conclusions

In conclusion, the diagnosis of GCT with secondary ABC is solely based on histopathological analysis. The clinical and radiological findings are nonspecific. It is imperative to obtain a precise tissue diagnosis in the preoperative period to plan appropriate treatment. A preoperative diagnosis of GCT may not change the treatment of combined GCT and ABC in the patella, but a concomitant malignant pathology in ABC if missed may have grave consequence.
